# Efflux-mediated fluoroquinolone resistance in the multidrug-resistant *Pseudomonas aeruginosa* clinical isolate PA7: identification of a novel MexS variant involved in upregulation of the *mexEF-oprN* multidrug efflux operon

**DOI:** 10.3389/fmicb.2015.00008

**Published:** 2015-01-21

**Authors:** Yuji Morita, Junko Tomida, Yoshiaki Kawamura

**Affiliations:** Department of Microbiology, School of Pharmacy, Aichi Gakuin UniversityNagoya, Japan

**Keywords:** *Pseudomonas aeruginosa*, efflux, mexXY-oprA, mexEF-oprN, mexS

## Abstract

The emergence of multidrug-resistant *Pseudomonas aeruginosa* has become a serious problem in medical settings. *P. aeruginosa* clinical isolate PA7 is resistant to fluoroquinolones, aminoglycosides, and most β-lactams but not imipenem. In this study, enhanced efflux-mediated fluoroquinolone resistance of PA7 was shown to reflect increased expression of two resistance nodulation cell division (RND) -type multidrug efflux operons, *mexEF-oprN* and *mexXY-oprA*. Such a clinical isolate has rarely been reported because MexEF-OprN-overproducing mutants often increase susceptibility to aminoglycosides apparently owing to impairment of the MexXY system. A mutant of PA7 lacking three RND-type multidrug efflux operons (*mexAB-oprM*, *mexEF-oprN*, and *mexXY-oprA)* was susceptible to all anti-pseudomonas agents we tested, supporting an idea that these RND-type multidrug efflux transporters are molecular targets to overcome multidrug resistance in *P. aeruginosa*. *mexEF-oprN*-upregulation in *P. aeruginosa* PA7 was shown due to a MexS variant harboring the Valine-155 amino acid residue. This is the first genetic evidence shown that a MexS variant causes *mexEF-oprN*-upregulation in *P. aeruginosa* clinical isolates.

## Introduction

*Pseudomonas aeruginosa* is a metabolically versatile bacterium that can cause a wide range of severe opportunistic infections in patients with serious underlying medical conditions (Gellatly and Hancock, 2013). Infections caused by *P. aeruginosa* often are hard to treat; inappropriate chemotherapy readily selects multidrug-resistant *P. aeruginosa* against which very few agents are effective (Morita et al., 2014; Poole, 2014). This so-called “antibiotic resistance crisis” has been compounded by the lagging in antibiotic discovery and development programs occurred in recent years, and is jeopardizing the essential role played by antibiotics in current medical practices (Rossolini et al., 2014). To combat this organism, it is very useful to understand its antimicrobial resistance mechanisms (see reviews such as Breidenstein et al., 2011; Poole, 2011; Morita et al., 2015).

The complete genome sequence of the multiresistant *P. aeruginosa* PA7 has been determined (Roy et al., 2010). The sequence of this strain, which exhibits resistance to fluoroquinolones (FQs), aminoglycosides, and various β-lactams but is susceptible to carbapenems (imipenem), includes typical FQ-resistance mutations in *gyrA* (Thr83Ile) and *parC* (Ser87Leu) (Roy et al., 2010). PA7 has additionally acquired a mutated *aacA4* gene, the product of which (AAC(6′)-II) endows the strain with aminoglycoside (gentamicin and tobramycin) resistance (Roy et al., 2010). This clinical isolate is amenable to the construction of gene knock-out mutations, thereby facilitating the analysis of molecular mechanisms of multidrug antimicrobial resistance in this isolate. Previously we showed that the effect of the modifying enzyme is enhanced by the MexXY resistance nodulation cell division (RND) -type multidrug efflux system, especially in the presence of divalent cations, providing high-level aminoglycoside resistance in *P. aeruginosa* (Morita et al., 2012b). This observation emphasizes the importance of the MexXY multidrug efflux system for aminoglycoside resistance in multidrug-resistant *P. aeruginosa* clinical isolates (Morita et al., 2012a).

Four members of the RND family of multidrug efflux systems, MexAB-OprM, MexCD-OprJ, MexEF-OprN, and MexXY have been implicated in FQ resistance in *P. aeruginosa* clinical isolates (Poole, 2011). As in other organisms, these RND family drug efflux transporters in *P. aeruginosa* operates as three-component pumps with an RND cytoplasmic membrane (CM) protein (MexB, MexD, MexF, and MexY) linked to an outer membrane channel-forming protein (OprM, OprJ, and OprN) by a CM-tethered periplasmic protein (MexA, MexC, MexE, and MexX) (Poole, 2013). Intriguingly PA7 possesses the *mexXY-oprA* multidrug efflux operon of which *oprA* is missing in most *P. aeruginosa* strains (Roy et al., 2010). We showed that the MexXY can utilize either the OprA or OprM as an outer membrane component of the tripartite efflux pump (Morita et al., 2012b). Multidrug-resistant *P. aeruginosa* clinical isolates, including carbapenem-resistant *P. aeruginosa*, often have been reported to be MexXY and/or MexAB-OprM overproducers (e.g., Hocquet et al., 2007; Cabot et al., 2011; Fuste et al., 2013; Khuntayaporn et al., 2013; Vatcheva-Dobrevska et al., 2013). FQ efflux is mediated by the MexAB-OprM system constitutively produced at moderate levels in wild-type *P. aeruginosa* PAO1 (Morita et al., 2001; Poole, 2013). Expression of *mexAB-oprM* is controlled directly or indirectly by three repressors encoded by the *mexR*, *nalC* and *nalD* genes, with inactivating mutations in any of these resulting in increased *mexAB-oprM* expression (Poole, 2011, 2013). In addition, *nfxB* and *nfxC* mutants (which overproduce components of the MexCD-OprJ and MexEF-OprN systems, respectively) are well-known as efflux-mediated FQ-resistant mutants of laboratory and clinical isolates of *P. aeruginosa*, although *nfxB* mutations appear to be rare in clinical settings (Poole, 2011). *nfxC* mutants also show increased resistance to carbapenems such as imipenem, not because MexEF-OprN accommodates these agents but because of a coordinated, MexT-dependent reduction of OprD production in such mutants (Poole, 2011, 2013). Hyperexpression of *mexEF-orpN* (and reduction in *oprD* expression) is also seen in laboratory isolates disrupted in the *mexS* gene, which encodes a putative oxidoreductase (a.k.a *qrh*) of unknown function (Sobel et al., 2005; Lamarche and Deziel, 2011). There are, however, no reports (to our knowledge) with experimental evidence of *mexEF-oprN*-expressing clinical isolates harboring mutations in *mexS* (Poole, 2013), although some studies suggested possible mutation of *mexS* in *nfxC*-type clinical isolates (e.g., Llanes et al., 2011; Fournier et al., 2013). The incidence of MexXY overproducers among clinical isolates has shown to be linked to the use of ciprofloxacin (Hocquet et al., 2008) and MexXY overproducers have been often reported to be one of major phenotypes in multidrug resistant clinical isolates (Morita et al., 2012a; Poole, 2013). However, MexXY-expressing FQ-resistance or its contribution to FQ-resistance in clinical settings has not well been studied compared to the other three pumps. In this study, we used reverse genetics to investigate the role of efflux-mediated FQ resistance in multidrug-resistant *P. aeruginosa* clinical isolate PA7.

## Materials and methods

### Bacterial strains, plasmids, and growth conditions

The bacterial strains and plasmids used in this study are listed in Table 1. Bacterial cells were grown in Luria (L) broth and on L agar (1.5%) under aerobic conditions at 37°C as previously described, unless otherwise indicated, with antibiotics as necessary (Morita et al., 2010). Bacterial growth was quantified by measuring the optical density at 600 nm on an Ultrospec 2100 Pro Spectrophotometer (GE Healthcare Corp., Tokyo, Japan), unless otherwise indicated. The plasmids pEX18Tc (Hoang et al., 1998), pYM101 (Morita et al., 2010), and their derivatives were maintained and selected using medium supplemented with 2.5-10 μg tetracycline ml^−1^ for *E. coli* or 50–150 μg tetracycline ml^−1^ for *P. aeruginosa*. The plasmids pUCP20T (Schweizer et al., 1996) and pFLP2 (Hoang et al., 1998) and their derivatives were maintained and selected using medium supplemented with 100 μg ampicillin ml^−1^ for *E. coli* or 200 μg carbenicillin ml^−1^ for *P. aeruginosa*.

**Table 1:** **Bacterial strains and plasmids**.

**Strains or plasmids**	**Relevant characteristics**	**References**
***E. COLI***		
DH5α (PAGU^g^121)	For recombinant DNA manipulation	Morita et al., 2010
KAM3 (PAGU^g^846)	For recombinant DNA manipulation	Morita et al., 1998
S17-1 (PAGU^g^856)	For conjugational transfer	Morita et al., 2006
***P. AERUGINOSA***		
PAO1 (PAGU 974)	Wild type	Morita et al., 2006
PA7 (PAGU 1498)	Multi-resistant clinical isolate	Roy et al., 2010
PAGU^g^1565	PA7 Δ*mexXY-oprA*	Morita et al., 2012b
PAGU^g^1603	PA7 Δ*mexAB-oprM*	Morita et al., 2012b
PAGU^g^1748	PA7 Δ*mexEF-oprN*	This study
PAGU^g^1641	PA7 Δ*mexXY-oprA* Δ*mexAB-oprM*	Morita et al., 2012b
PAGU^g^1751	PA7 Δ*mexXY-oprA* Δ*mexEF-oprN*	This study
PAGU^g^1753	PA7 Δ*mexAB-oprM* Δ*mexEF-oprN*	This study
PAGU^g^1756	PA7 Δ*mexXY-oprA* Δ*mexAB-oprM* Δ*mexEF-oprN*	This study
PAGU^g^1793	PA7 Δ*mexS*	This study
PAGU^g^1789	PA7 Δ*mexT*	This study
PAGU^g^1867	PA7 *mexS(V155A)*	This study
DSM 1128 (PAGU 1504)	Taxonomically PA7-related strain, wild-type	Kiewitz and Tummler, 2000
K2153 (PAGU 1741)	Clinical isolate	Sobel et al., 2003
K2376 (PAGU^g^1834)	K2153 Δ*mexS*	Fetar et al., 2011
PAGU^g^1850	K2153 Δ*mexS attB::lacI^q^*-P_*T*7_	This study
PAGU^g^1851	K2153 Δ*mexS attB::lacI^q^*-P_*T*7_-*mexS_PA7_*	This study
PAGU^g^1844	K2153 Δ*mexS attB::lacI^q^*-P_*T*7_-*mexS*_*DSM* 1128_	This study
PAGU^g^1854	K2153 Δ*mexS* pUCP20T	This study
PAGU^g^1855	K2153 Δ*mexS* pUCP20T::*mexS_PA7_*	This study
PAGU^g^1856	K2153 Δ*mexS* pUCP20T::*mexS*_*DSM* 1128_	This study
PAGU^g^1835	K2153 Δ*mexT*	Fetar et al., 2011
PAGU^g^1836	K2153 Δ*mexF*	Fetar et al., 2011
K2942 (PAGU^g^1837)	K2153 Δ*mexS* Δ*mexT*	Fetar et al., 2011
PAGU^g^1852	K2153 Δ*mexS* Δ*mexT attB::lacI^q^-P_*T*7_*	This study
PAGU^g^1846	K2153 Δ*mexS* Δ*mexT attB::lacI^q^-P_*T*7_-mexT_PA7_*	This study
PAGU^g^1845	K2153 Δ*mexS* Δ*mexT attB::lacI^q^-P_*T*7_-mexT*_*DSM* 1128_	This study
**PLASMID**		
pEX18Tc	Broad-host-range gene replacement vector	Hoang et al., 1998
pYM133	pEX18Tc::Δ*mexEF-oprN*	This study
pYM134	pEX18Tc::Δ*mexS*	This study
pYM135	pEX18Tc::Δ*mexT*	This study
pYM136	pEX18Tc::Δ*mexST*	This study
pYM137	pEX18Tc::*mexS_PA7_*	This study
pYM138	pEX18Tc::*mexS_PA7_(V155A)*	This study
pYM101	*P. aeruginosa* chromosome integration vector	Morita et al., 2010
pYM139	pYM101::*mexS_PA7_*	This study
pYM140	pYM101::*mexS*_*DSM* 1128_	This study
pYM141	pYM101::*mexT_PA7_*	This study
pYM142	pYM101::*mexT*_*DSM* 1128_	This study
pUCP20T	Broad-host-range cloning vector	Schweizer et al., 1996
pYM143	pUCP20T::*mexS_PA7_*	This study
pYM144	pUCP20T::*mexS*_*DSM* 1128_	This study
pFLP2	Flp recombinase-producing plasmid	Hoang et al., 1998

### Molecular biology techniques

Plasmid DNA isolation from *E. coli*, DNA purification, measuring DNA concentration, DNA digestion with restriction enzymes, DNA dephosphorylation, DNA ligation, isolation of chromosomal DNA from *P. aeruginosa*, PCR conditions, nucleotide sequencing, competent cell preparation from *E. coli*, transformation of *E. coli*, and transfer of plasmids into *P. aeruginosa* via conjugation were performed as described previously (Morita et al., 2010), unless otherwise indicated. DNA sequences and amino acid sequences were analyzed through 2012 to 2013 with Pseudomonas Genome Database (Winsor et al., 2011), Basic Local Alignment Search Tool (BLAST) (Boratyn et al., 2013), SWISS-MODEL (Bordoli et al., 2009), Sorting Tolerant From Intolerant (SIFT) BLink (Kumar et al., 2009), and the software DNASIS Pro (Ver. 2.1; Hitachi, Japan).

### Cloning of *mexS* and *mexT* from *P. aeruginosa*

*mexS* and *mexT* from *P. aeruginosa* (without endogenous promoters) were amplified by PCR using the primers listed in Table 2. The purified *mexS* PCR product, digested with BamHI and HindIII, was cloned into similarly digested, dephosphorylated pUCP20T and pYM101, yielding pUCP20T::*mexS* and pYM101::*mexS*. The purified *mexT* PCR product, digested with EcoRI and BamHI, was cloned into similarly digested, dephosphorylated pUCP20T and pYM101, yielding pUCP20T::*mexT* and pYM101::*mexT*.

**Table 2:** **Primers used in this study**.

**Primer**	**Sequence(5′–3′)**	**Purpose**	**References**
EcoRI-*mexE*_PA7_-UF	GAT CGA ATT CTG GCC TCG GGG GAA ATC T	*mexEF-oprN* genes disruption of *P. aeruginosa* PA7	This study
BamHI-*mexE*_PA7_-UR	CTG AGG ATC CAT GCT TGA CTC CGC CAG TC	*mexEF-oprN* genes disruption of *P. aeruginosa* PA7	This study
BamHI-*oprN*_PA7_-DF	CTG AGG ATC CTG AAC CGG CTA TCC CCG G	*mexEF-oprN* genes disruption of *P. aeruginosa* PA7	This study
XhoI-*oprN*_PA7_-DR	GAT CCT CGA GCG CCG ACA GCG ATT GCC A	*mexEF-oprN* genes disruption of *P. aeruginosa* PA7	This study
BamHI-*mexS*_PA7_-F	GAT CGG ATC CGA TGC ACT GCA GAG GTT TGC	*mexS* gene cloning of *P. aeruginosa* PA7 and DSM 1128	This study
HindIII-*mexS*_PA7_-R	CTA GAA GCT TCA ATC GGC GAC GTG GAT	*mexS* gene cloning of *P. aeruginosa* PA7 and DSM 1128	This study
EcoRI-*mexS*_PA7_-UF	GCT AGA ATT CAG TTC GTC GGT GTA GCT GA	*mexS* gene disruption of *P. aeruginosa* PA7	This study
BamHI-*mexS*_PA7_-UR	CTA GGG ATC CGG ACA TCG CAA ACC TCT G	*mexS* gene disruption of *P. aeruginosa* PA7	This study
BamHI*-mexS*_PA7_-DF	GCT AGG ATC CGT CGC CGA TTG AGG ACG AC	*mexS* gene disruption of *P. aeruginosa* PA7	This study
HindIII-*mexS*_PA7_-DR	CTA GAA GCT TAG TAC ATC CAC GCG CAC CT	*mexS* gene disruption of *P. aeruginosa* PA7	This study
EcoRI-*mexT*_PA7_-F	GAT CGA ATT CCC CTG GAA ACG AGG AAC	*mexT* gene cloning of *P. aeruginosa* PA7 and DSM 1128	This study
BamHI-*mexT*_PA7_-R	GAT CGG ATC CTC AGA GGC TGT CCG GGT C	*mexT* gene cloning of *P. aeruginosa* PA7 and DSM 1128	This study
SacI-*mexT*_PA7_-UF	GCT AGA GCT CTG GAA ACG ATC ACC CGC G	*mexT* gene disruption of *P. aeruginosa* PA7	This study
BamHI-*mexT*_PA7_-UR	CTG AGG ATC CAT GGC GTT CCT CGT TTC C	*mexT* gene disruption of *P. aeruginosa* PA7	This study
BamHI-*mexT*_PA7_-DF	GCT AGG ATC CGG ACA GCC TCT GAG TCA TCC ACG	*mexT* gene disruption of *P. aeruginosa* PA7	This study
HindIII-*mexT*_PA7_-DR	CTA GAA GCT TGC GAC CGC CGC CCT GGC TT	*mexT* gene disruption of *P. aeruginosa* PA7	This study
*mexS_PA7_(V155A)*-F	CTG ATC ACC GAG GCG GCG CGC ATG TAC GGG CC	*mexS*(V155A) site directed mutagenesis of *P. aeruginosa* PA7	This study
mexS_PA7_(V155A)-R	GGC CCG TAC ATG CGC GCC GCC TCG GTG ATC AG	*mexS*(V155A) site directed mutagenesis of *P. aeruginosa* PA7	This study
*mexTS* intergenic seq	GCA CCA GGA CTT CCC CTG	DNA sequencing of *mexT-mexS* intergenic region	This study
*uvrD*-F	ATCGACTTCTCCGAGCTGCTG	RT-PCR for *uvrD* gene of *P. aeruginosa*	Morita et al., 2012b
*uvrD*-R	CTGGAACTCGTCCACCAGGAT	RT-PCR for *uvrD* gene of *P. aeruginosa*	Morita et al., 2012b
*mexE*-F (RT)	CCA CCC TGA TCA AGG ACG AAG	RT-PCR for *mexE* gene of *P. aeruginosa*	This study
*mexE*-R (RT)	CGG TAG ACG GTC TTG TTG TCG	RT-PCR for *mexE* gene of *P. aeruginosa*	This study
*mexS*-F (RT)	AGG GCG TCA ATG TCA TCC TC	RT-PCR for *mexS* gene of *P. aeruginosa*	This study
*mexS*-R (RT)	CTG CAG GTG CTT CTT GAA CG	RT-PCR for *mexS* gene of *P. aeruginosa*	This study
*mexT*-F (RT)	TAT TGA TGC CGA ACC TGC TG	RT-PCR for *mexT* gene of *P. aeruginosa*	This study
*mexT*-R (RT)	GGA GGA TCT TCG GCT TGC TG	RT-PCR for *mexT* gene of *P. aeruginosa*	This study
*oprD*-F (RT)	ATT GCA CTG GCG GTT TCC	RT-PCR for *oprD* gene of *P. aeruginosa*	This study
*oprD*-R (RT)	ATG AAC CCC TTC GCT TCG	RT-PCR for *oprD* gene of *P. aeruginosa*	This study
*mexA*-F (RT)	GAC AAC GCT ATG CAA CGA ACG	RT-PCR for *mexA* gene of *P. aeruginosa*	This study
*mexA*-R (RT)	AGC TCG GTA TTC AGC GTC ACC	RT-PCR for *mexA* gene of *P. aeruginosa*	This study

### Construction of in-frame deletion mutants from *P. aeruginosa*

In-frame deletion mutants of *mexEF-oprN*, *mexS*, and *mexT* from *P. aeruginosa* PA7 and its derivatives were constructed using the previously described *sacB*-based strategy (Morita et al., 2006, 2010). The plasmids and resulting *P. aeruginosa* mutants are listed in Table 1, while the primer pairs are listed in Table 2. The selection concentrations of tetracycline for the first homologous recombination event were adjusted to reflect the endogenous tetracycline MICs for the *P. aeruginosa* strains. These constructs were confirmed by colony PCR.

### Construction of the ΦCTX-based site-specific integrants in *P. aeruginosa*

For gene complementation experiments in *P. aeruginosa*, Φ CTX phage-based site-specific integrants were constructed using the integration-proficient, tightly controlled expression vector pYM101 and associated techniques (conjugative transfer, gene replacement at the chromosomal *attB* site, and curing of the unwanted plasmid backbone from the chromosome via the pFLP2-encoded Flp recombinase) as previously described (Morita et al., 2010).

### Site-directed mutagenesis of *mexS*_*PA7*_ in the chromosome of *P. aeruginosa* PA7

An amino acid substitution (Val155Ala) mutation was introduced into the *mexS_PA7_* gene of the insert in the plasmid pUC20T::*mexS_PA7_*. Site-directed mutagenesis within a target plasmid was carried out according to Geiser et al. (2001) and Morita et al. (2009). A 50-μl mixture consisting of 50 ng of plasmid DNA, 0.25 μM of each mutagenic primer pair (Table 2), 0.2 mM each deoxynucleoside triphosphate, 1 mM MgSO_4_, 2.5 U of KOD Hot Start DNA polymerase -Plus- Ver.2 (TOYOBO Co. Ltd., Osaka, Japan), 1× KOD buffer, and 4.0% (vol/vol) dimethyl sulfoxide was heated to 94°C for 2 min followed by 18 cycles of 0.5 min at 94°C, 1 min at 60°C, and 5 min at 68°C. The resulting DNA products were purified as above, digested overnight with 10 U DpnI (Roche Diagnostics K.K., Tokyo, Japan) to eliminate template plasmid), and used to transform *E. coli* DH5α. Plasmids were recovered from individual transformants, and the *mexS* insert was sequenced to identify plasmids bearing the desired mutation. The *mexS* insert carrying the mutation was digested with BamHI and HindIII and cloned into similarly digested and dephosphorylated pEX18Tc to yield pEX18Tc::*mexS_PA7_(V155A)*. The *mexS_PA7_(V155A)* mutation was gene replaced onto the chromosome of *P. aeruginosa* PA7 using the previously described *sacB*-based strategy (Morita et al., 2009, 2012b).

### Antibiotic susceptibility assay

The susceptibility of *P. aeruginosa* to antimicrobial agents in cation-adjusted Mueller–Hinton broth was assessed using the 2-fold serial microtiter broth dilution method described previously (Morita et al., 2012b). Minimal inhibitory concentrations (MICs) were defined as the lowest concentration of antibiotic resulting in visible inhibition of growth after 18 h of incubation at 37°C. MICs for the Φ CTX-based site-specific integrants were determined in the presence of the inducer 5 mM isopropyl-β-D-1-thiogalactopyranoside (IPTG). The categorization in susceptible, intermediate, and resistant was performed according to the interpretive standards of the Clinical and Laboratory Standards Institute (CLSI) (Patel et al., 2011).

### Antimicrobial agents

Amikacin, ampicillin, carbenicillin, chloramphenicol, ciprofloxacin, norfloxacin, and tetracycline were purchased from Wako Pure Chemicals Industries, Ltd (Osaka, Japan). Moxifloxacin and levofloxacin were purchased from LKT Laboratories, Inc. (St. Paul, MN, USA). Imipenem/cilastatin was purchased from Sandoz K.K. (Tokyo, Japan).

### Real-time quantitative reverse transcriptase (qRT)-PCR

Overnight cultures of *P. aeruginosa* strains were diluted 1:100 in 10 ml, incubated with vigorous shaking at 37°C for 3–4 h, and harvested. Total RNA was stabilized with the RNA Protect Bacteria Reagent (Qiagen) and isolated with the RNeasy Mini Kit (Qiagen). The RNA samples were further treated with RQ1 RNase-Free DNase (Promega) and purified by using the RNeasy Mini Kit (Qiagen). Real-time qRT-PCR was performed with primer pairs internal to *uvrD*, *mexE*, *mexS*, *mexT*, and *oprD* (Table 2) using the One Step SYBR PrimeScript RT-PCR kit II (TaKaRa) in a Thermal Cycler Dice real-time system (TaKaRa). The transcript levels of the target gene in a given strain were normalized to levels of *uvrD* and expressed as a ratio (fold change) to that observed in the parental PA7 strain. Gene expression values were calculated based on triplicate experiments.

### Nucleotide sequence accession number

The nucleotide sequences of DNA regions containing *mexT* and *mexS* in *P. aeruginosa* DSM 1128 have been deposited in GenBank/EMBL/DDBJ with the accession number AB889539.

## Results

### Contribution of RND multidrug efflux systems to FQ resistance in *P. aeruginosa* PA7

FQ resistance in *P. aeruginosa* PA7 was compared with the two FQ-susceptible strains, PAO1 (the standard laboratory strain) and DSM 1128 (a strain taxonomically related to PA7) (Morita et al., 2012b). The MICs of the FQs (ciprofloxacin, levofloxacin, moxifloxacin, and norfloxacin) for *P. aeruginosa* PA7, PAO1, and DSM 1128 are shown in Table 3. *P. aeruginosa* PA7 exhibited increased resistance (ca. 256-fold) to these FQs compared to *P. aeruginosa* PAO1 and DSM 1128. *P. aeruginosa* DSM 1128 showed the same level of resistance to the FQs as *P. aeruginosa* PAO1. Based on the interpretive standards of the CLSI (Patel et al., 2011), *P. aeruginosa* PA7 was considered highly resistant to these FQs. This observation is not surprising, because *P. aeruginosa* PA7 possesses typical target mutations, one in *gyrA* (Thr83Ile) and one in *parC* (Ser87Leu), well-known to be associated with FQ resistance (Lomovskaya et al., 1999). However, target mutations alone are not sufficient to explain high-level FQ resistance in *P. aeruginosa*; the additional effect of up-regulation of one of the four RND efflux pumps (MexXY-OprA, MexAB-OprM, MexCD-OprJ, or MexEF-OprN) is necessary (Lomovskaya et al., 1999; Bruchmann et al., 2013).

**Table 3:** **Contribution of three RND efflux pumps to antimicrobial resistance of *P. aeruginosa* PA7**.

**Strain**	**Genotype**	**MIC (μg/ml) of**						
		**CIP**	**NOR**	**LVX**	**MXF**	**AMK**	**CHL**	**IPM**
PA7 (PAGU 1498)	Parent	64	128	128	256	32	1024	2
PAGU^g^1565	PA7 Δ*mexXY-oprA*	32	64	64	128	1	1024	2
PAGU^g^1603	PA7 Δ*mexAB-oprM*	64	128	64	256	32	1024	2
PAGU^g^1748	PA7 Δ*mexEF-oprN*	32	64	64	256	32	128	2
PAGU^g^1641	PA7 Δ*mexXY-oprA* Δ*mexAB-oprM*	32	64	32	64	1	1024	2
PAGU^g^1751	PA7 Δ*mexXY-oprA* Δ*mexEF-oprN*	8	16	8	32	1	256	2
PAGU^g^1753	PA7 Δ*mexAB-oprM* Δ*mexEF-oprN*	32	64	32	128	32	16	2
PAGU^g^1756	PA7 Δ*mexXY-oprA* Δ*mexAB-oprM* Δ*mexEF-oprN*	0.5	0.5	0.5	2	1	4	2
DSM 1128 (PAGU 1504)	Wild type	0.125	0.25	0.25	1	0.5	32	1
PAO1 (PAGU 974)	Wild type	0.125	0.25	0.25	1	0.5	32	1

*AMK, amikacin; CHL, chloramphenicol; CIP, ciprofloxacin; IPM, imipenem; LVX, levofloxacin; MXF, moxifloxacin; NOR, norfloxacin*.

Previously we showed that the Δ*mexXY-oprA* Δ*mexAB-oprM* double mutant of PA7 was slightly more susceptible (ca. 2- to 4-fold) to ciprofloxacin, while retaining high-level resistance (MIC = 32 μg/ml) to this antibiotic when compared to its parental strain (Morita et al., 2012b). That observation was inconsistent with data, derived via similar genetic analyses, in which a PAO1-derived Δ*oprM gyrA parC* triple mutant (i.e., a strain deleted for *oprM* and carrying the target mutations) exhibited increased susceptibility (64-fold) to levofloxacin compared to an isogenic MexAB-OprM overexpressing (*nalB*) mutant, while exhibiting an MIC (0.5 μg/ml) of levofloxacin similar to that of the wild-type PAO1 (0.25 μg/ml) (Lomovskaya et al., 1999). We noted that deletion of *oprM* in PAO1 was expected to inactivate any efflux pumps that require OprM as an outer membrane factor (e.g., MexAB-OprM, MexXY-OprM, MexVW-OprM) (Morita et al., 2001; Li et al., 2003). We therefore hypothesized that overexpression of other RND pumps such as MexCD-OprJ and MexEF-OprN was compensating for the effects of the *mexAB-oprM mexXY-oprA* double deficiency in PA7. As a first step in testing our hypothesis, we examined the primary sequences of the efflux system-encoding genes of PA7. Sequence analysis revealed the presence of *mexCD-oprJ* (PSPA7_0540- 0539- 0538) and *mexEF-oprN* (PSPA7_2745-2744-2743) homologs in *P. aeruginosa* PA7 (Roy et al., 2010), with predicted amino acid lengths (in PA7) and amino acid sequence identity and similarity [respectively; via BLAST of PA7 vs. PAO1-UW (Winsor et al., 2011)] as follows: MexC (387 aa; 88 and 95%), MexD (1043 aa; 95 and 98%,), OprJ (475 aa; 89 and 93%); MexE (414 aa; 99 and 99%), MexF (1062 aa; 99 and 99%), and OprN (472 aa; 97 and 99%). As a second step in testing our hypothesis, we examined the regulation of these genes in PA7. qRT-PCR showed that expression of *mexE* was increased (ca. 22-fold) in PA7 compared to the wild-type strain DSM 1128. This observation indicated that FQ resistance in *P. aeruginosa* can be mediated by overexpression of the *mexEF-oprN* operon, in addition to the previously reported overexpression of *mexXY-oprA* due to mutation of the local repressor gene (*mexZ*) (Morita et al., 2012b).

We also observed that a Δ*mexXY-oprA* Δ*mexAB-oprM* Δ*mexEF-oprN* triple mutant derived from PA7 exhibited increased susceptibility to FQs (32–128-fold) compared to an isogenic Δ*mexXY-oprA* Δ*mexAB-oprM* double mutant, while exhibiting only mild elevation of FQ MIC (0.5–2 μg/ml) compared to wild-type PAO1 and DSM 1128 (0.125–1 μg/ml) (Table 3). Moreover, the triple mutant was much more sensitive (512-fold) to chloramphenicol than the double mutant, a further indicator of MexEF-OprN-mediated resistance (Sobel et al., 2005) (Table 3). We excluded a role for overexpression of *mexCD-oprJ* in PA7, as judged by the susceptibilities of the mutant (Δ*mexXY-oprA* Δ*mexAB-oprM* Δ*mexEF-oprN* construct) to not only FQs but also to tetracycline and chloramphenicol, which are substrates of MexCD-OprJ (Poole et al., 1996).

To clarify the roles of MexXY-OprA, MexAB-OprM and MexEF-OprN multidrug efflux pumps in the FQ resistance of *P. aeruginosa* PA7, we constructed a series of deletion mutants of the three *mex* operons and examined their drug susceptibility profiles (Table 3). We observed that the Δ*mexXY-oprA* Δ*mexEF-oprN* double deletion mutant was more susceptible (4-fold) than the Δ*mexAB-oprM* Δ*mexXY-oprA* double mutant and the Δ*mexAB-oprM* Δ*mexEF-oprN* double mutant (Table 3). These data suggested that the MexXY-OprA and MexEF-OprN systems make similar contributions to FQ resistance in *P. aeruginosa* PA7, each having an effect larger than that of MexAB-OprM. The apparent modest MexAB-OprM contribution to resistance of FQs and chloramphenicol in PA7 [PA7 Δ*mexXY-oprA* Δ*mexEF-oprN* vs. PA7 Δ*mexXY-oprA* Δ*mexAB-oprM* Δ*mexEF-oprN* (Table 3)] is typically observed in wild type *P. aeruginosa* such as PAO1 already shown in the previous results (e.g., PAO1 Δ*mexXY* vs. PAO1 Δ*mexXY* Δ*mexAB-oprM*) (e.g., Morita et al., 2001), implying that *mexAB-oprM* is expressed at moderate levels in PA7. We concluded that high-level FQ resistance in *P. aerguinosa* PA7 was due to overproduction of MexXY-OprA and MexEF-OprN, in addition to the typical target mutations.

### Molecular mechanisms of *mexEF-oprN*-upregulation in *P. aeruginosa* PA7

The *mexEF-oprN* operon is quiescent in wild-type *P. aeruginosa* cells grown under standard laboratory conditions. In contrast, this operon is expressed in so-called *nfxC* mutants and in mutants defective in the *mex*S gene (previously known as *qrh*), a locus that encodes a putative oxidoreductase of as yet unknown function (Poole, 2013). Expression of *mexEF-oprN* is regulated by a transcriptional activator, MexT, a LysR family regulator (Kohler et al., 1999; Poole, 2013). *mexT* occurs upstream of *mexEF-oprN* and downstream of *mexS*, the latter gene also positively regulated by MexT (Kohler et al., 1999). Unusually, many so-called wild type strains possess inactive MexT (e.g., 8 bp insertion in *mexT* present in some PAO1 strains) (Maseda et al., 2000; Poole, 2013). The induction of *mexEF-oprN* contributes to multidrug resistance, albeit against a rather narrow range of antimicrobials including FQs, trimethoprim, and chloramphenicol (Poole, 2013). The enhanced resistance to imipenem of *nfxC* mutants or *mexS* deficient mutants results not from *mexEF-oprN* expression but the concomitant decrease in the level of outer membrane protein OprD (Poole, 2013), because OprD is an imipenem channel and serves as the primary route of entry of this antibiotic in *P. aeruginosa* (Trias and Nikaido, 1990). However, *P. aeruginosa* PA7 was reported to be susceptible to carbapenems, with MICs of 2 and 1 μg/ml for imipenem and meropenem, respectively (Roy et al., 2010). This observation of carbapenem susceptibility is somewhat paradoxical, given that a typical *nfxC* mutant or a *mexS* deficient mutant is expected to exhibit carbapenem resistance due to a coordinate, MexT-dependent reduction of OprD levels. We found that *P. aeruginosa* PA7 exhibited slight but reproducible elevation (2-fold) of resistance to imipenem compared to wild-type *P. aeruginosa* strains PAO1 and DSM 1128 (Table 4). Deletion of *mexS* in a PA7 background resulted in slightly increased expression of *mexE* (2-fold) and increased resistance to imipenem (4-fold) compared to PA7. Thus, PA7 appeared to exhibit susceptibility intermediate between that of wild type and a typical *nfxC* mutant. Deletion of *mexT* in a PA7 background resulted in drastically (>200-fold) decreased expression of *mexE* and *mexS* compared to PA7 (Table 4). Notably, expression of *mexS* in PA7 Δ*mexT* was more than 96-fold reduced compared to the wild-type DSM 1128 and PA7 *mexS (V155A)*. This observation was consistent with a previous report (using β-galacotosidase reporter assays) indicating that *mexS* (*qrh*) is constitutively expressed at a moderate level (Kohler et al., 1999; Poole, 2013).

**Table 4:** **Relationship between antimicrobial resistance and *mexS-mexT*-mediated *mexEF-oprN* expression in *P. aeruginosa***.

**Strains**	**Genotype**	**MIC (μg/ml) of**			**Relative mRNA level**		
		**CIP**	**CHL**	**IPM**	***mexE***	***mexS***	***mexT***
PA7 (PAGU 1498)	Parent	128	1024	2	1	1	1
PAGU^g^1793	PA7 Δ*mexS*	>128	>1024	8	2.0	nd	0.84
PAGU^g^1867	PA7 *mexS*(V155A)	64	256	1	0.053	0.57	0.74
PAGU^g^1789	PA7 Δ*mexT*	64	128	1	<0.005	<0.005	nd
DSM 1128 (PAGU 1504)	Wild type	0.125	32	1	0.046	0.48	1.1
PAO1 (PAGU 974)	Wild type	0.125	32	1	nd	nd	nd

*CHL, chloramphenicol; CIP, ciprofloxacin; IPM, imipenem; nd, not done*.

We therefore determined the nucleotide sequences (ca. 2.4 kb) upstream of the *mexEF-oprN* operon in *P. aeruginosa* DSM 1128 (accession no. AB889539). This interval corresponds to the operon's cognate regulatory region, and includes the *mexS* and *mexT* loci. Comparison to the corresponding sequences from *P. aeruginosa* PA7 (data not shown) revealed that MexS_PA7_ is encoded as an A155V variant, and MexT_PA7_ as an A256T variant, compared to the DSM 1128 genome. In contrast, alanine-155 of MexS and alanine-256 of MexT are conserved among multiple laboratory and clinical strains, including PAO1, PA14, PAK, and K2153 (Sobel et al., 2005; Jin et al., 2011; Lamarche and Deziel, 2011). To test the function of the *mexS_PA7_* and *mexT_PA7_* loci, we amplified and cloned the individual *mexS* and *mexT* genes (without endogenous promoters) from *P. aeruginosa* PA7 and DSM 1128, and expressed the relevant genes in *P. aeruginosa* K2153 Δ*mexS* (Fetar et al., 2011) for a *mexS* complementation test or in K2153 Δ*mexS* Δ*mexT* (Sobel et al., 2005; Fetar et al., 2011) for a *mexT* complementaton test (Table 5). [As demonstrated by other researchers, *P. aeruginosa* K2153 and its derivatives are useful for the analysis of *mexEF-oprN*-dependent antimicrobial resistance as regulated by the MexT activator and MexS function (Sobel et al., 2005; Fetar et al., 2011)]. Interestingly, introduction of *mexS*_*DSM* 1128_ or *mexS_PA7_* into the chromosome of K2153 Δ*mexS* did not provide complementation of Δ*mexS* (Table 5). These data contrast previous instances in which we observed *attB*-site (i.e., single-copy) complementation for several other genes (Morita et al., 2010, 2012b). Presumably, in the present studies, failure to complement reflected insufficient *mexS* expression from the P_*T*7_ promoter in PAGU^g^1844, given that MexS_DSM 1128_ is expected to be functional. Introduction of pUCP20T::*mexS_DSM 1228_* into *P. aeruginosa* K2153 Δ*mexS* yielded MICs identical to those of the K2153 *mexS^+^* parent; introduction of pUCP20T::*mexS_PA7_* yielded parent-like resistance to imipenem, but only partially restored resistance to ciprofloxacin and chloramphenicol (Table 5). Taken together, these results suggested that MexS_PA7_ provides reduced function compared to MexS_DSM 1228_. Additionally it appears that the high levels of *mexS* expression are required to overcome the *nfxC*-type antimicrobial resistance.

**Table 5:** **Functional characterization of *mexS* and *mexT* from *P. aeruginosa* PA7 and DSM 1128**.

**Strains**	**Genotype**	**MIC (μg/ml)**		
		**CIP**	**CHL**	**IPM**
K2153 (PAGU 1741)	Parent	0.5	64	2
K2376 (PAGU^g^1834)	Δ*mexS*	2	2048	8
PAGU^g^1850	Δ*mexS attB::lacI^q^*-P_T7_	2	2048	8
PAGU^g^1851	Δ*mexS attB::lacI^q^*-P_*T*7_*-mexS_PA7_*	2	2048	8
PAGU^g^1844	Δ*mexS attB::lacI^q^*-P_*T*7_*-mexS*_*DSM* 1128_	2	1024	8
PAGU^g^1854	Δ*mexS* pUCP20T	2	2048	8
PAGU^g^1855	Δ*mexS* pUCP20T::*mexS_PA7_*	1	256	4
PAGU^g^1856	Δ*mexS* pUCP20T::*mexS*_*DSM* 1128_	0.5	64	2
K2942 (PAGU^g^1837)	D*mexS* Δ*mexT*	0.5	64	2
PAGU^g^1852	Δ*mexS* Δ*mexT attB::lacI^q^*-P_*T*7_	0.5	64	2
PAGU^g^1846	Δ*mexS* Δ*mexT attB::lacI^q^-P*_*T*7_*-mexT_PA7_*	2	2048	8
PAGU^g^1845	Δ*mexS* Δ*mexT attB::lacI^q^-P*_*T*7_*-mexT*_*DSM* 1128_	2	2048	8

*CHL, chloramphenicol; CIP, ciprofloxacin; IPM, imipenem*.

*5 mM IPTG was added to allow expression controlled by the P_*T*7_ promoter*.

Slightly increased expression of *mexS* was observed in *P. aeruginosa* PA7 compared to *P. aeruginosa* DSM 1128 (ca. 2-fold), while expression levels of *mexT* in *P. aeruginosa* PA7 were similar to those in *P. aeruginosa* DSM 1128 (c.a. 0.9-fold) (Table 4). Introduction of *mexT*_*DSM* 1128_ or *mexT_PA7_* into the chromosome of K2153 Δ*mexS* Δ*mexT* yielded the *nfxC* phenotype (Table 5), suggesting that the A256T substitution in MexT is not a primary reason for overexpression of *mexEF-oprN* in *P. aeruginosa* PA7. Using site-specific mutagenesis, we altered the plasmid-borne *mexS_PA7_* locus to encode a V155A version of the protein and replaced the endogenous PA7 chromosomal locus with the mutated gene. The PA7 *mexS_PA7_(V155A)* strain showed decreased *mexE* expression (0.053-fold compared to that of the PA7 parent), a value similar to that seen in DSM 1128 (0.034-fold compared to that of PA7) (Table 4). The PA7 *mexS_PA7_(V155A)* strain also showed decreased MICs for chloramphenicol (0.25-fold) (Table 4). Taken together with the complementation experiments, these data strongly suggested that the A155V substitution in MexS_PA7_ is the primary reason for increased expression of *mexEF-oprN* and increased antimicrobial resistance in the PA7 strain.

## Discussion

In this study, the multidrug-resistant clinical isolate PA7 was shown to exhibit increased expression of *mexEF-oprN* as well as *mexXY-oprA*. Although multidrug-resistant *P. aeruginosa* clinical isolates have often been reported to be MexXY overproducers (Morita et al., 2012a), clinical strains of *P. aeruginosa* overproducing MexEF-OprN and MexXY(-OprA) efflux pumps simultaneously have rarely been reported. In fact, PA7 was not a typical *nfxC* mutant (i.e., a MexEF-OprN overproducer), and instead expressed intermediate levels of *mexEF-oprN*. We assume that simultaneous overproduction of MexEF-OprN and MexXY(-OprA) impairs *P. aeruginosa* growth, based on our observation that our PA7 Δ*mexS* construct (i.e., a simultaneous overproducer of MexEF-OprN and MexXY-OprA compared to the PA7 parent) was unstable even on L agar plates: colonies of the construct exhibited a non-uniform phenotype during growth on plates (data not shown). This observation is consistent with the increased susceptibility to aminoglycosides previously observed in MexEF-OprN-overproducing *nfxC* mutants, apparently owing to impairment of the MexXY system (Sobel et al., 2005).

Valine-155 of MexS_PA7_ also was shown as the likely primary reason for increased production of MexEF-OprN in PA7. With the exception of *mexS_PA7_*, sequenced *P. aeruginosa mexS* genes (including those from PAO1, K2153, PA14, PAK, and DSM 1128) encode proteins with an alanine at residue 155 (Sobel et al., 2005; Jin et al., 2011; Lamarche and Deziel, 2011). The Ala155Val substitution is predicted by the SIFT algorithm (Kumar et al., 2009) not to affect the protein's function (data not shown). We hypothesize that MexS_PA7_ retains function, albeit with decreased activity and/or altered regulation (e.g., allostery), compared with the other MexS orthologs. In fact, there were few differences among the whole structures of MexS_PA7_, MexS_PAO1_, and MexS_DSM 1128_ models developed by using the SWISS-MODEL program (data not shown) (Bordoli et al., 2009). MexS is a member of the cd08268: MDR2 family of the Conserved Domains Database (CDD) of the National Center for Biotechnology Information (NCBI) (Fargier et al., 2012) and alanine-155 corresponds to one of the putative NAD(P) binding sites featured in the MDR2 family.

In addition to antibiotic resistance, an *nfxC*-type mutation has been linked to reduced levels of homoserine lactone-dependent quorum sensing (QS) -regulated virulence factors, including pyocyanin, elastase, rhamnolipids, and Pseudomonas Quinolone Signal (PQS), and to reduced expression of type-III secretion system (TTSS) effector proteins (Kohler et al., 2001; Linares et al., 2005). QS is a cell-to-cell communication mechanism employing diffusible signal molecules (Jimenez et al., 2012), and the TTSS is a mechanism by which bacterial pathogens can deliver effectors directly into the cytoplasm of eukaryotic host cells (Hauser, 2009). We found that PA7 had reduced level of pyocyanin production, rhamnolipid production, and swarming activity compared to DSM 1128 and PAO1 (data not shown), consistent with the typical *nfxC* mutant phenotype (Jin et al., 2011). However, PA7 derivatives including PA7 *mexS_PA7_(V155A)* also showed almost the same activities of the QS regulated virulence factors with PA7 (data not shown), which suggests that the impaired QS-related phenotype is not derived from the *nfxC*-like phenotype in PA7. We presume that this lack of correlation reflects the absence from PA7 of TTSS-encoding genes and of the *mvfR* (*pqsR*) gene, which is known to encode a LysR-type transcriptional regulator that modulates the expression of multiple QS-regulated virulence factors (Deziel et al., 2005; Roy et al., 2010). These deficiencies might be the source of the *mexS* mutation and increased *mexEF-oprN* expression under oxidative stress, sulfide stress, or nitrosative stress (Juhas et al., 2004; Fetar et al., 2011; Fargier et al., 2012).

### Conflict of interest statement

The authors declare that the research was conducted in the absence of any commercial or financial relationships that could be construed as a potential conflict of interest.
